# Involvement of paraspeckle components in viral infections

**DOI:** 10.1080/19491034.2024.2350178

**Published:** 2024-05-08

**Authors:** Romane Milcamps, Thomas Michiels

**Affiliations:** Université catholique de Louvain, de Duve Institute, Brussels, Belgium

**Keywords:** Interferon, internal ribosome entry site (IRES), long non-coding RNA, NEAT1, paraspeckles, SFPQ, viral replication, viruses

## Abstract

Paraspeckles are non-membranous subnuclear bodies, formed through the interaction between the architectural long non-coding RNA (lncRNA) nuclear paraspeckle assembly transcript 1 (NEAT1) and specific RNA-binding proteins, including the three *Drosophila Behavior/Human Splicing* (DBHS) family members (PSPC1 (Paraspeckle Component 1), SFPQ (Splicing Factor Proline and Glutamine Rich) and NONO (Non-POU domain-containing octamer-binding protein)). Paraspeckle components were found to impact viral infections through various mechanisms, such as induction of antiviral gene expression, IRES-mediated translation, or viral mRNA polyadenylation. A complex involving NEAT1 RNA and paraspeckle proteins was also found to modulate interferon gene transcription after nuclear DNA sensing, through the activation of the cGAS-STING axis. This review aims to provide an overview on how these elements actively contribute to the dynamics of viral infections.

## Introduction

Paraspeckles, initially identified in 2002 as a novel nuclear domain within mammalian cells, represent dynamic subnuclear bodies with dimensions typically ranging from 0.5 to 1.0 µm [1]. Over the years, extensive research allowed the identification of their structural components and highlighted their involvement in various cellular functions.

Paraspeckles are formed through the interaction between Nuclear Enriched Abundant Transcript 1 (NEAT1), an architectural long non-coding RNA (lncRNA), and several RNA-binding proteins, including members of the Drosophila Behavior/Human Splicing (DBHS) family. Paraspeckles are membraneless subnuclear bodies that result from liquid-liquid phase separation (LLPS), a process facilitated by intrinsically disordered regions (IDRs) of the RNA-binding proteins included in their composition [2].

While the full extent of their cellular functions remains to be defined, paraspeckles and their components have been ascribed a number of functions, which have been proposed to serve as global stress sensors [3]. Expression of NEAT1 RNA as well as paraspeckle assembly and disassembly depend on stress factors such as the transcription factors p53 and ATF2 or on the activity of the MAP kinase pathway.

Paraspeckles act at the interface of mitosis, DNA repair, development and cancer [4–7]. They are also connected to mitochondrial stress and were reported to modulate innate immune responses including the inflammatory response of macrophages [8,9]. Their involvement in antiviral defense mechanisms has recently garnered significant attention.

Many of these functions depend on the ability of paraspeckle components to regulate gene expression via the sequestration of RNAs and specific proteins [10–13].

In the field of virology, it is well-established that viruses are obligate intracellular pathogens that rely heavily on the host cell machinery for replication and propagation. To date, the understanding of the complex interaction between viruses and their hosts has focused mainly on protein-based interactions. Recent breakthroughs however emphasized the significant involvement of lncRNA in host antiviral responses [14–18]. For instance, NeST RNA (cleanup Salmonella not Theiler’s), a mouse lncRNA, has been shown to regulate interferon gamma expression [19] and the cellular lncRNA NRON, a repressor of the transcription factor NFAT (Nuclear Factor of Activated T cells), was found to be modulated during HIV-1 infection [20,21].

NEAT1, the scaffold lncRNA of paraspeckles, has emerged as another player in viral infections. Additionally, other paraspeckle components have been implicated in responses to viral infections, highlighting the complex interplay between paraspeckles and viral pathogens. These discoveries underscore the broader significance of paraspeckles in regulating gene expression across various cellular processes, including their role in responding to viral infections.

Before giving an overview of current knowledge regarding the involvement of paraspeckle components in viral infections, we will shortly introduce paraspeckle formation, the specific components that make up these structures and their cellular functions. This will contribute to a deeper comprehension of the intricate roles played by paraspeckles in the context of viral infections.

## Paraspeckle formation

Virus-inducible non-coding RNA (VINC), more recently referred to as NEAT1, was first detected in the brain of mice infected with either Japanese encephalitis virus or Rabies virus [22]. It plays a crucial role in the formation of paraspeckles, as reviewed in [23], by interacting with specific RNA-binding proteins, including members of the *Drosophila Behavior/Human Splicing* (DBHS) family. Paraspeckles, which are non-membranous subnuclear bodies, are present in the interchromatin space of nearly all cell lines and tissues, except for human embryonic stem cells [10].

### Long non-coding RNA NEAT1

**Figure 1.**
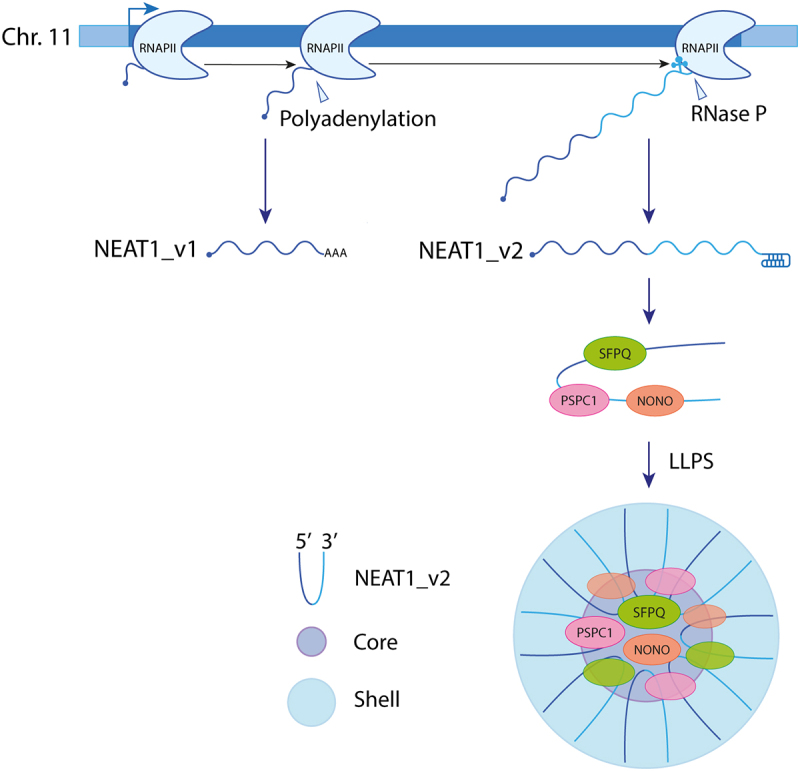
Formation of paraspeckles. RNA polymerase II transcribes two overlapping isoforms of NEAT1, namely NEAT1_v1 and NEAT1_v2, from the same gene locus on human chromosome 11 through alternative 3’ processing. Unlike the short NEAT1_v1 isoform, the long NEAT1_v2 lacks a polyA tail. NEAT1_v2 interacts with paraspeckle proteins, including members of the DBHS family (SFPQ, NONO, and PSPC1) to form an initial ribonucleoprotein. This RNP undergoes oligomerization and matures into a paraspeckle. Within the paraspeckle, NEAT1_v2 RNAs adopt a U-shaped structure with their 5’ and 3’ ends located in the peripheral region (shell) and their middle parts located in the central core.

Two NEAT1 isoforms, NEAT1_v1 and NEAT1_v2, with distinct 3’ end processing, are transcribed by RNA polymerase II (RNAPII) from a common promoter on human chromosome 11. While the short NEAT1_v1 isoform is polyadenylated, the long NEAT1_v2 acquires a stable triple-helix structure after 3' end cleavage by Ribonuclease P (Rnase P), preventing its degradation [24,25]. Concurrently with its biogenesis, NEAT1_v2, which is the essential isoform for paraspeckle formation [25,26], associates with specific proteins to form an individual NEAT1_v2 ribonucleoprotein (RNP). This RNP is subsequently assembled into a larger paraspeckle structure comprising approximately 50 NEAT1_2 RNPs [27], through liquid – liquid phase separation (LLPS) [2,23]. Within paraspeckles, NEAT1_v2 adopts a U-shaped structure with its 5’ and 3’ ends located in the peripheral region (shell) and its middle part located in the central core [28] (Figure 1).

### Paraspeckle proteins

The first paraspeckle proteins identified were the three *Drosophila Behavior/Human Splicing* (DBHS) family members (PSPC1 (Paraspeckle Component 1), SFPQ (Splicing Factor Proline- and Glutamine-Rich) and NONO (Non-POU domain-containing octamer-binding protein)), the RNA-binding protein 14 (RBM14), and the cleavage and polyadenylation specificity factor subunit 6 (CPSF6) [1,11,29]. Currently, more than 40 proteins, most of which exhibit RNA-binding properties, have been recognized as paraspeckle constituents based on their colocalization with SFPQ [25]. The essential factors for the formation of paraspeckles are listed in Table 1 [2,25].

**Table 1.** Essential factors for paraspeckle formation.

Factors	Function in paraspeckles	Molecular function
NEAT1_v2	Structural scaffold	Architectural lncRNA
RNA polymerase II	NEAT1 transcription	RNA polymerase
NONO	NEAT1_2 stability	RNA-binding protein
SFPQ	NEAT1_2 stability	RNA-binding protein
RBM14	NEAT1_2 stability	RNA-binding protein
HNRNPK	Inhibition of NEAT1_v1 polyadenylation	RNA-binding protein
RNase P	NEAT1_v2 3’-end processing	Endoribonuclease
FUS	Paraspeckle assembly	RNA-binding protein
DZAP1	Paraspeckle assembly	RNA-binding protein
HNRNPH3	Paraspeckle assembly	RNA-binding protein
SWI/SNF complex	Paraspeckle assembly	Chromatin remodeling

**Figure 2.**
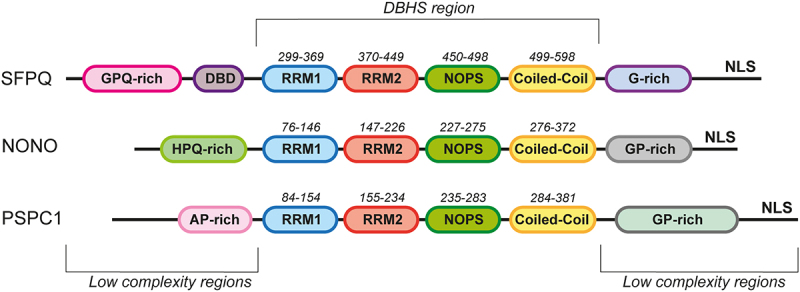
Structure of the DBHS proteins. Schematic representation of the domain organization of the human DBHS family proteins. The core conserved region (DBHS region) contains tandem RNA recognition motifs (RRM1 (blue) and RRM2 (red)), a NonA/paraspeckle domain (NOPS (green)), and a coiled-coil domain (yellow). The *N*- and C-terminal regions of the proteins are low-complexity regions. The DNA-binding domain in SFPQ is designated as DBD (purple). NLS is a nuclear localization signal.

The DBHS proteins, encompassing three members (PSPC1, NONO, and SFPQ), constitute a family of nucleic acid-binding proteins that operate as homo- or hetero-dimers. Structurally, these proteins feature a conserved core region, called DBHS region (\~300aa), containing tandem RNA recognition motifs (RRMs) involved in RNA and DNA interaction, a NonA/paraspeckle domain (NOPS) involved in DBHS dimerization, and a C-terminal coiled-coil which facilitates dimerization and oligomerization. Beyond these shared regions, family members display variations in both length and sequence complexity. The DBHS region is flanked by low-complexity regions that drive liquid-liquid phase separation during paraspeckle formation. DBHS proteins also display a C-terminal nuclear localization signal (NLS) [4] (Figure 2).

In addition to their essential role in paraspeckle formation, these proteins actively participate in various nuclear processes, such as DNA repair and regulation of gene expression (reviewed in [4]). These proteins have been implicated in various aspects of RNA metabolism, including transcription, pre-mRNA splicing, and RNA transport. They have also been shown to participate in the regulation of alternative splicing and to interact with RNA polymerase II and other splicing factors [4,5].

In summary, the DBHS family proteins are multifunctional nuclear proteins that play a key role in the complex machinery of cellular functions, impacting DNA repair, RNA metabolism and gene expression.

## Cellular functions of paraspeckles

The cellular functions of paraspeckles are not yet fully understood, but they are believed to be stress-induced structures that regulate gene expression by sequestering edited RNAs and proteins. The first proposed mechanism is the nuclear retention of hyperedited RNAs derived from transcribed inverted repeat Alu elements. Nuclear RNA duplexes are targeted for adenosine-to-inosine editing by adenosine deaminase (ADAR), preventing their export to the cytoplasm as they interact with paraspeckle proteins, particularly NONO, which exhibit a high affinity for inosine-containing RNAs [10–13]. The second proposed function of paraspeckles is the sequestration of a variety of nuclear proteins, including DBHS proteins. This action reduces the accessible nuclear reservoir of these factors, thereby impacting the expression of their target genes [30–32]. For example, SFPQ has been shown to function as a transcription factor, promoting ADARB2 expression [30] while inhibiting IL-8 expression [31]. Stress-induced paraspeckle assembly increases SFPQ sequestration within paraspeckles, thus inhibiting the activities of free SFPQ. Consequently, this leads to the downregulation of ADARB2 expression and the upregulation of IL-8 expression. Interestingly, ADARB2 lacks catalytic activity and is therefore considered as a competitor of other ADAR proteins.

## Involvement of paraspeckle components in viral infections

The host innate immune response constitutes the first line of defense against viral infection. It acts through the activation of pattern recognition receptors (PRRs), which rapidly recognize specific viral components such as viral RNA or DNA, as well as replication intermediates. In response to this activation, type I interferons (IFNs) and other pro-inflammatory cytokines are produced to trigger an antiviral response in adjacent and distant cells, rendering them refractory to infection [33]. As a growing number of studies point to the involvement of paraspeckle components in the regulation of gene expression in diverse cellular processes, including responses to viral infections, this article aims to provide an overview of how these elements actively contribute to the dynamics of viral infections. Table 2 provides a list of viruses for which paraspeckle components have been documented to play a role in the infection process.

**Table 2.** Involvement of paraspeckle components in viral infections.

Viral genome	Family	Virus	Paraspeckle components	Effects	Ref
ssRNA(+)	*Picornaviridae*	Encephalomyocarditis virus	SFPQ	Viral translation (ITAF)	[34]
		Poliovirus	NONO	Viral replication	[35]
		Coxsackievirus B3	SFPQ	Viral translation (ITAF)	[36]
		Rhinovirus	SFPQ (cleavage)	Viral replication	[37]
	*Flaviviridae*	Dengue virus	NEAT1 downregulation	Correlation with the development of a severe dengue phenotype	[38]
		Zika virus	NEAT1 downregulation	?	[39]
	*Coronaviridae*	SARS-CoV-2	SFPQ	Viral replication	[40]
	*Togaviridae*	Sindbis virus	SFPQ/NONO/PSPC1/RBM14/MATR3	Viral replication	[41]
ssRNA(−)	*Orthomyxoviridae*	Influenza virus A	SFPQ	Viral mRNA polyadenylation	[42]
			NEAT1 upregulation RBM14	Antiviral gene expression Viral replication	[31] [43]
	*Bunyaviridae*	Hantavirus	NEAT1 upregulation (RIG-I/IRF7)	Antiviral gene expression	[44]
	*Kolmioviridae*	Hepatitis delta virus	NEAT1 upregulation	Viral replication	[45]
			SFPQ/NONO/PSPC1	Viral replication	[45–48]
RNA/DNA	*Retroviridae*	Human immunodeficiency virus 1	NEAT1 upregulation	Reverse transcription, integration, maintenance of unspliced viral mRNA levels	[49]
dsDNA	*Herpesviridae*	Herpes simplex 1 virus	NEAT1 upregulation (STAT3)	Viral replication	[50]
		Epstein-Barr virus	SFPQ/NONO/RBM14	Viral gene expression and lytic cycle modulation	[51]
		Pseudorabies virus	NONO	Antiviral gene expression	[52]

### Induction of NEAT1 expression by viruses

Virus infection exerts significant influence on the cellular transcriptome, causing changes in the expression of long non-coding RNAs (lncRNAs) such as NEAT1. To date, two different pathways were shown to be involved in the induction of NEAT1 expression in response to viral infection.

The first pathway inducing NEAT1 expression is the RIG-I/IRF7 pathway, discovered in Hantavirus (HTNV)-infected cells [44]. Upon increased expression, NEAT1 suppressed the transcriptional inhibitory effects of SFPQ by translocating SFPQ to paraspeckles, thus promoting the expression of several genes, including those encoding DDX60 – an essential factor for both RIG-I activation and viral RNA degradation [53] – and RIG-I itself. This process supports robust interferon responses and suppresses HTNV infection [44]. Secondly, NEAT1 was reported to be upregulated in a STAT3-dependent manner in cells infected with Herpes simplex virus 1 (HSV-1) [50], thus promoting paraspeckle formation.

Several other viruses were shown to upregulate NEAT1 expression, likely through one of the two aforementioned pathways. For example, influenza virus infection triggers a four-fold increase in NEAT1 RNA expression, which increases paraspeckle formation in infected cells. As a consequence, the paraspeckle protein SFPQ which physiologically downregulates the transcription of several genes by binding to their promoters, relocates to paraspeckles, thus relieving the transcriptional repression of genes such as the one encoding interleukin-8 (IL-8) [31].

### Regulation of IRES-dependent translation

Initially identified in picornaviruses, internal ribosome entry sites (IRESs) are characterized by specific RNA sequences that interact with IRES trans-acting factors (ITAFs). Together, they facilitate cap-independent ribosome recruitment to initiate protein synthesis [54]. While most cellular mRNAs typically undergo translation using cap-dependent processes, it is now acknowledged that under specific conditions, translation of some genes can occur independently of the cap structure, suggesting a shift in the cell’s translation mode, from cap-dependent to cap-independent [55].

**Figure 3.**
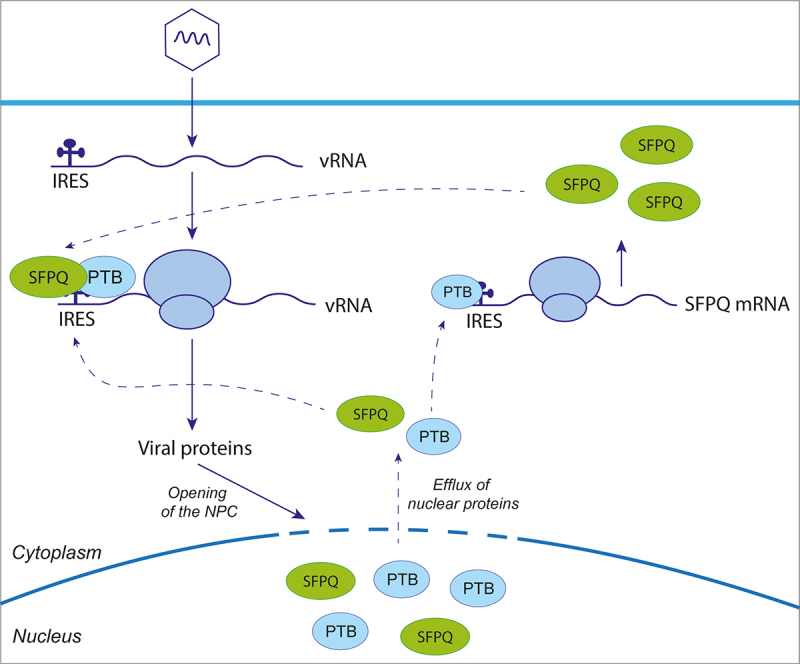
SFPQ can act as an IRES trans-activating factor. Upon viral infection, cellular factors recognized as ITAFs, including SPFQ and PTB, relocalize from nucleus to cytoplasm, promoting viral IRES-mediated translation. SFPQ also has an IRES element in its mRNA, which is stimulated by PTB. Consequently, the cellular level of SFPQ is maintained, even under condition where cap-dependent translation is inhibited, thus providing a favorable environment for viral IRES-mediated translation.

Coxsackievirus B3 (CVB3) and Encephalomyocarditis virus (EMCV) are non-enveloped positive single-stranded RNA viruses of the *Picornaviridae* family, belonging to the *Enterovirus* and *Cardiovirus* genera respectively. Their genome replication occurs in membranous complexes in the cytoplasm of infected cells, and translation is IRES-mediated. Polypyrimidine tract-binding protein (PTB) is recognized as an ITAF that promotes IRES-mediated translation of many picornavirus genomes [56], including those of CVB3 and EMCV. PTB also acts as an ITAF for the translation of SFPQ, which relies on an IRES [36,57]. Intriguingly, both CVB3 and EMCV are also believed to exploit SFPQ as an ITAF [34,36]. PTB and SFPQ have a typical nuclear localization. Interestingly, entero- and cardioviruses were shown to induce nucleocytoplasmic trafficking disruption by nucleoporin degradation or hyperphosphorylation [58–60], thus allowing translocation of PTB and SFPQ from the nucleus to the cytoplasm, where they bind viral RNA to promote IRES-mediated translation [34,36] (Figure 3).

This highlights two important mechanisms that are exploited by viruses during shut down of cap-dependent translation. First, viral infection induces relocalization of nuclear ITAFs to the cytoplasm, thereby promoting increased viral RNA translation. Second, ITAFs like SFPQ also undergo IRES-mediated translation to maintain their levels in infected cells.

### Facilitation of viral mRNA polyadenylation

The genome of Influenza A virus (IAV) consists of eight segments of negative-sense viral RNA. Each of these segments is bound by multiple copies of the nucleoprotein and by the heterotrimeric (PB1-PB2-PA) viral RNA-dependent RNA polymerase, forming viral ribonucleoprotein (vRNP) complexes. Proteomic analysis of purified polymerase expressed in human HEK-293T cells identified SFPQ as a polymerase-associated cellular protein [61]. In the nucleus, where IAV replicates, SFPQ has been shown to increase the polyadenylation efficiency of primary and secondary viral transcripts [42]. It has been hypothesized that SFPQ might interact with both the viral polymerase and the viral polyadenylation signal within the vRNP. This interaction would enhance the polymerase ability to carry out polyadenylation of viral mRNAs by repeatedly copying the oligo U signal located near the 5’-terminus of the viral RNA template [62].

SFPQ is not the only paraspeckle protein that facilitates IAV replication. RBM14, another paraspeckle protein, has been described as a pro-viral factor for IAV, since its depletion reduced the virus yield [43].

### Regulation of viral replication

SFPQ has been reported to interact with various viruses and to influence their replication. For example, SFPQ undergoes specific cleavage by the viral protease 3 CD/3C of human rhinovirus 16 (HRV16). The resulting C-terminal fragment of SFPQ translocates from the nucleus to the cytoplasm, where it associates with HRV RNA [37]. It has been suggested that the truncated form of SFPQ promotes viral RNA stability or replication. SFPQ has also been identified as a binder of SARS-CoV-2 and Sindbis virus RNA, thereby promoting their replication. For both viruses, knockdown of SFPQ resulted in a decrease in vRNA levels [40,41].

NONO is another paraspeckle component that interacts with enterovirus RNA and in particular poliovirus (PV) RNA. As with SFPQ and rhinovirus, this interaction does not affect PV RNA translation but does influence viral replication, and more specifically the synthesis of the positive strand of viral RNA [35].

It has been reported that SFPQ [46] and NONO [47] bind to the two terminal stem-loop domains of Hepatitis delta virus (HDV) RNA, both in the plus- and the minus-RNA strands. Since these regions also encompass sites recognized by the RNA polymerase II (RNAPII) to initiate HDV RNA transcription [48], it was suggested that these proteins might have a role in RNAP II-mediated HDV replication. Besides SFPQ and NONO, PSPC1 has also been shown to associate with HDV RNA. It appears that these three paraspeckle proteins are important for HDV replication, as their knockdown significantly impairs HDV replication in HEK-293 cells [45].

**Figure 4.**
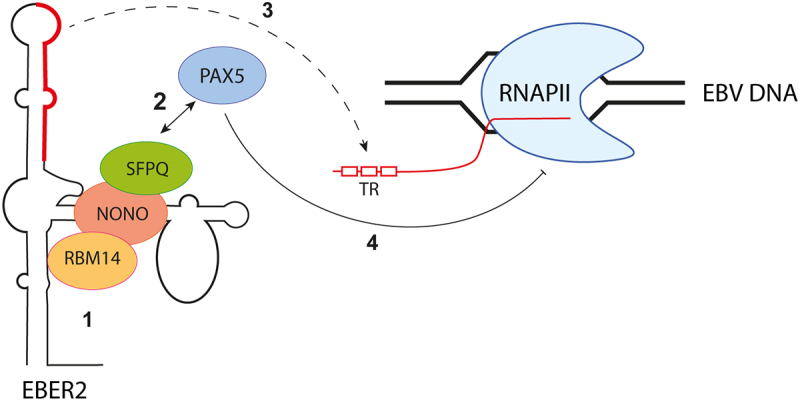
EBER2 interacts with PAX5 through paraspeckle proteins to regulate viral gene expression. The noncoding RNA EBER2, produced by EBV, interacts with SFPQ, NONO, and RBM14 (step 1) to facilitate its association with PAX5 (step 2). The recruitment of the EBER2-PAX5 complex is orchestrated through base-pairing interactions between EBER2 and the TR region, located in the first intron of the viral transcripts encoding latent membrane protein (LMP) (step 3). The EBER2-PAX5 complex exhibits asuppressive function, regulating the transcription of LMP genes (step 4).

Epstein-Barr virus (EBV), a member of the *Herpesviridae* family, employs yet another mechanism involving paraspeckle proteins to regulate its replication. EBV encodes non-coding RNAs known as EBV-encoded RNAs (EBERs). Among these RNAs, EBER2 interacts with the cellular transcription factor paired box protein 5 (PAX5) through paraspeckle proteins such as SFPQ, NONO, and RBM14 [51] (Figure 4). This complex is subsequently recruited, via base-pairing interactions, to a specific region of the viral genome, known as the TR region. The TR region is located in the first intron of the viral transcripts encoding latent membrane protein (LMP) 2A and 2B. By binding to this region, the EBER2-PAX5 complex downregulates the transcription of LMP genes [51,63]. EBV has thus hijacked the ability of paraspeckle proteins to interact with non-coding RNAs to control viral gene expression and modulate its lytic cycle.

The paraspeckle protein, PSPC1, was also shown to regulate herpesvirus replication, in the case of HSV-1. PSPC1 can interact with and recruit STAT3 to paraspeckles. There, STAT3 interacts with viral gene promoters including that of the gene encoding proteins which activate the lytic cycle such as ICP0 or thymidine kinase. This activation ultimately results in an increase in viral gene expression and viral replication. However, another paraspeckle protein, SFPQ, can compete with STAT3 for binding to viral gene promoters and thereby modulate the effects of STAT3 [50].

SFPQ and NONO were also shown to affect various stages of human immunodeficiency virus-1 (HIV-1) replication, including reverse transcription, integration, and the maintenance of unspliced viral mRNA levels. These activities of paraspeckle proteins on HIV were reviewed in [49].

### Induction of interferon expression by herpesviruses

Recently, paraspeckle components have been shown to exert positive effects on innate immunity by upregulating the expression of IFNs and other cytokines in response to herpesvirus infection. For example, NONO has been identified as an essential host factor in counteracting pseudorabies virus (PRV) infection, through activation of IFN gene transcription and, consequently, the expression of interferon-stimulated genes (ISGs) [52].

A novel ribonucleoprotein complex involving NEAT1 RNA and paraspeckle proteins has been identified as a regulator of foreign nuclear DNA sensing, in cells infected by Kaposi’s sarcoma-associated herpesvirus (KSHV). In this case, NEAT1, which is upregulated by infection, associates with HEXIM1, DNA-PK, and paraspeckle proteins (SFPQ, NONO, PSPC1, RBM14, and MATRIN3) to form a complex named HEXIM1-DNA-PK-paraspeckle components ribonucleoprotein(HDP-RNP) [64]. Upon non-self DNA detection, such as during KSHV infection, paraspeckle proteins like NONO would disengage from the complex, whereas the NEAT1, HEXIM1, DNA-PK containing complex would interact with cGAS and STING to induce the cGAS-STING pathway, thereby promoting IRF3-mediated activation of IFN gene transcription [64]. Interestingly, as a countermeasure, KSHV encodes ORF52 which targets cGAS and inhibits its activation. It was found that expression of ORF52 resulted in the loss of binding of HEXIM1 to cGAS, suggesting a novel viral strategy to avoid initiation of innate immune response through HDP-RNP [64].

## Discussion

The paradigm shift from a focus on protein-based interactions in the study of virus-host relationships to the recognition of the significant involvement of cellular regulatory RNA has been a crucial step forward. While only a small fraction of the genome is transcribed into protein-coding mRNAs, non-coding RNAs, including lncRNAs have emerged as key regulators of cellular processes, such as gene expression, organelle activity, and response to viral infections.

The formation of non-membranous subnuclear bodies known as paraspeckles, mediated by the architectural lncRNA NEAT1 and specific RNA-binding proteins, represents a prominent example of the structural and functional roles of lncRNAs. Paraspeckles act as dynamic cellular structures involved in the sequestration of specific RNA species and proteins, thereby modulating gene expression. Additionally, the recognition of paraspeckles as responders and regulators of the host defense in the innate immune response highlights their versatile role in combating invading pathogens.

**Figure 5.**
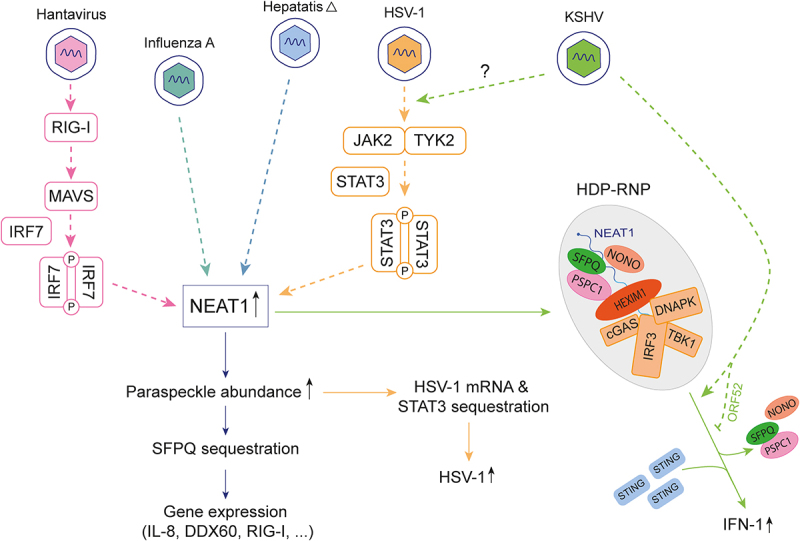
Paraspeckle components actively contribute to the dynamics of viral infections. Overexpression of NEAT1 in response to viral infection increases the assembly of paraspeckles, which exert antiviral effects by sequestering SFPQ, thus attenuating its transcriptional inhibitory effect on antiviral genes (IL-8, DDX60 and RIG-I) (light blue and pink). Conversely, paraspeckles have pro-viral effects for HSV-1, as STAT3 sequestration promotes increased HSV-1 replication (orange). Upon non-self DNA detection, paraspeckle proteins disengage from the NEAT1-HEXIM complex, which in turn activates the cGAS-STING pathway, culminating in activation of IFN gene transcription (green). KSHV encodes ORF52 which inhibits the formation of HDP-RNP complex.

Paraspeckle components do impact viral infections through various mechanisms (Figure 5). SFPQ, a key paraspeckle protein, acts as an internal ribosome entry site (IRES) trans-acting factor, influencing cap-independent ribosome recruitment during the translation of specific viral RNAs [34,36]. Paraspeckle proteins, including SFPQ, NONO and RBM14, interact with viral RNAs, impacting various stages of the viral life cycle, from replication [35,37,40,41,43] to mRNA polyadenylation [42]. Furthermore, paraspeckle components play a crucial role in bridging interactions between cellular and viral factors, as exemplified by their involvement in the regulation of the Epstein-Barr virus (EBV) lytic cycle through interaction with the noncoding RNA EBER2 [51]. Finally, NEAT1 appears as a dual regulator of viral infection, exhibiting both pro-viral and anti-viral effects depending on the context [31,38,39,44,45,49,50,64,65].

The link between paraspeckles and stress granules (SGs) adds a further layer of complexity to cellular stress responses. Paraspeckles and SGs, located in the nucleus and cytoplasm respectively, appear to share a significant overlap in their proteomes [66]. Notably, paraspeckle hyperassembly occurs under SG-induced stress, and the formation of visible SGs is crucial for initiating and maintaining stress-induced paraspeckle assembly [66]. Mechanistically, SGs can sequester paraspeckle negative regulators, attenuating their inhibitory effect on paraspeckles [66].

Finally, examining patients’ serum for differentially expressed cellular lncRNA could offer an early diagnostic tool for tracking viral disease progression. While in dengue virus infection, the decreased level of NEAT1 correlates with the development of a severe dengue phenotype [38], in SARS-CoV-2 infection, NEAT1 has been associated with the cytokine storm [67–69].

In conclusion, the study of lncRNAs has provided a deeper understanding of the regulatory mechanisms governing cellular processes, especially in the context of viral infection. Unraveling the detailed mechanisms underlying the interactions between paraspeckle components and various viruses not only provides insights for future research but also opens avenues for potential diagnostic or therapeutic interventions targeting these complex host-virus relationships.

## Data Availability

Data sharing is not applicable to this article as no new data were created or analyzed in this study.
